# The German Pharmacological Society during the Nazi regime (1933–1945) and its resurrection after World War II

**DOI:** 10.1007/s00210-025-04605-x

**Published:** 2025-11-01

**Authors:** Nina Sophie Goebels, Christine Wolters, Roland Seifert

**Affiliations:** 1 Institute of Pharmacology, Hannover Medical School, 30625 Hannover, Germany; 2 Institute for Ethics, History and Philosophy of Medicine, Hannover Medical School, 30625 Hannover, Germany

**Keywords:** History of pharmacology, National Socialism, Pharmacological chair holders, Biographies, DGPT

## Abstract

This study examines the German Pharmacological Society (DGP) during the Nazi Regime (1933-1945) and its resurrection after World War II. The analysis is based on DGPT documents stored in the archives of Hannover Medical School and published in *Naunyn-Schmiedebergs Arch Pharmacol*. The minutes of board meetings were a key source of information. Selected individuals who played an important role during this timeare briefly presented biographically and scientifically, and their positions are explained. These individuals are Behrend Behrens, Otto Riesser, Hellmut Weese, Wolfgang Heubner, Ferdinand Flury, Otto Loewi, and Otto Krayer. The study focuses on the DGP’s treatment of its Jewish members—both during and after World War II. It examines the extent to which the professional association in post-war Germany took a position on its actions or inaction at that time. In 1931/32, 18% of the DGP members were Jewish. We identified 23 additional DGP members persecuted by the Nazis not yet known through previous studies. They will be analyzed and honored in detail in a future study. In order to classify the behavior of the DGP, a brief comparison with other professional associations (pediatrics and gynecology) is also made. Our study shows that the DGP has not come to terms with its past behavior during the Nazi era until recently. Although Jewish members were not explicitly excluded from the professional association, which distinguishes the DGP from other professional associations, there are indications of vehement representatives of Nazi ideology (e.g., Ferdinand Flury) within the professional association. The DGP board never officially contradicted these openly professed supporters of Nazism after the Nazis came to power. There are also indications of a certain political calculation to quickly establish ties with the scientific community, which ultimately succeeded. This context explains that Otto Riesser, persecuted during the Nazi era, was elected as the first DGP chairman after the war. Although Prof. Dr. Ursula Gundert-Remy issued an official apology in 2014 for the behavior of the DGP toward Jewish members, it remains unclear why this acknowledgment came so late.

## Introduction

The German Pharmacological Society, originally known as the *Deutsche Pharmakologische Gesellschaft* (DPhG), was founded on September 24, 1920, during the 86th Assembly of German Natural Scientists and Physicians held in Bad Nauheim. The first board consisted of the pharmacologists Arthur Heffter, Walther Straub, Josef Schüller, Rudolf Gottlieb, Alexander Ellinger, and Hans Horst Meyer (Muscholl 1995). This foundation laid the groundwork for what would eventually become the *Deutsche Gesellschaft für Experimentelle und Klinische Pharmakologie und Toxikologie* (DGPT), the current German Society for Experimental and Clinical Pharmacology and Toxicology.

The DGPT as it exists today emerged from the merger of three specialist organizations: the German Society for Pharmacology (DGP), the German Society for Clinical Pharmacology and Therapy (*Deutsche Gesellschaft für Klinische Pharmakologie und Therapie e.V.*, DGKliPha), and the Society for Toxicology (GT). This structural consolidation was formally reflected in the society’s adoption of its current name in 1993. Today, the DGPT represents a multidisciplinary society and counts approximately 2500 members across the fields of experimental pharmacology, clinical pharmacology, and toxicology. The aim of the DGPT is to promote the teaching of the subject at universities and colleges, pharmacological and toxicological research, and the international representation of the three specialist societies. The scientific organ of the society is Naunyn Schmiedeberg’s Archives of Pharmacology (DGPT 2022).

With the exception of the Second World War, DGP conferences were held almost every year. The last conference before the outbreak of the war was the 14th conference, which took place in Berlin from April 24 to 28, 1938. This was followed by a break of nine years until the 15th conference was held in Hamburg from 22 to 24 August 1947. Following this conference, the first preliminary meeting on the re-founding of the DGP took place on October 7, 1947, at the Pharmacological Institute of the University of Frankfurt. Six members were elected as the new board. These members were August Wilhelm Forst, Ernst Frey, Fritz Külz, Josef Schüller, Otto Riesser, and Hellmut Weese. Riesser was elected as First Chairman and Weese as Managing Director. The minutes of the meeting state that the board first had to be approved by the military government, as it was to be made up of “gentlemen [sic] from the British and American occupation zones.” This was made easier by the fact that all six board members were politically unencumbered. If possible, the society should include members from the English, American, and French occupation zones. It would have been unlikely that the name “Deutsche Pharmakologische Gesellschaft” would have been approved, so “Pharmakologische Gesellschaft (Walter Straub)” or “Walter-Straub-Gesellschaft” were suggested as alternatives. It was important for those present to identify the German nationality in relation to foreign pharmacological societies. The resolutions of this meeting were not to be enforced until Prof. Behrend Behrens and Prof. Wolfgang Heubner, as the last managing director, had approved them (Protokoll der Vorstandssitzung of October 7, 1947, ArchMHH, Dep. 13, 2). The society was officially registered on July 29, 1949, as the “Deutsche Pharmakologische Gesellschaft, Sitz Düsseldorf” (Protokoll der Vorstandssitzung of September 1, 1949, ArchMHH, Dep.13, 2).

The attitude of former members of the DGP in the post-war period (1945–1949) and the period of National Socialism (1933–1945) has hardly been investigated to date. One possible reason for this is probably the poor state of records within pharmacology. Nevertheless, the topic is an important research desideratum. The aim of this work is therefore to reconstruct this development at this time as well as possible with the aid of other types of files.

## Methods and materials

### Data collection and data sources

The basis of the data presented here is the Dep. 13 (“DGPT Archive”) in the archive of the Hannover Medical School. The special focus of this study was on the board minutes (“Protokoll der Vorstandssitzung”) from the years 1947–1960. Board minutes from the years before 1947 have not survived. One reason for this could be the destruction of many pharmacological institutes, particularly the Pharmacological Institute in Berlin, whereby parts of the DGPT archive may have been lost. Another reason could be the destruction of files, correspondence and private estates to destroy possible evidence of the extent of support for National Socialism.

A bibliographical search of the most important personalities was carried out using selected book and internet sources. The publications of these personalities were analyzed using the database of the Naunyn–Schmiedeberg’s Archives of Pharmacology (https://link.springer.com/journal/210).

## Results and discussion

**Fig. 1:**
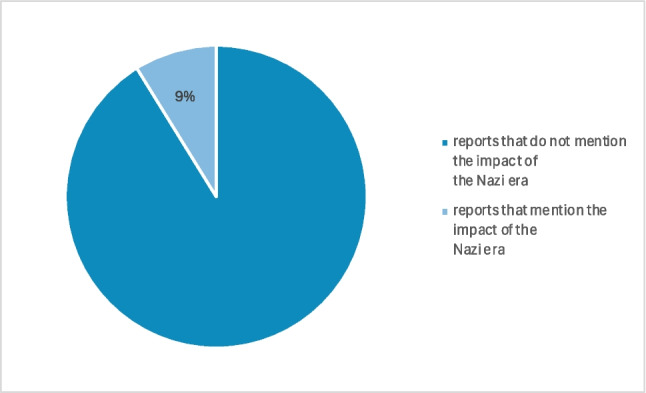
Proportion of reports relating to the Nazi era (institutes founded before 1945) (source: Philippu, A (2004): Geschichte und Wirken der pharmakologischen, klinisch-pharmakologischen und toxikologischen Institute im deutschsprachigen Raum, Band 1)

**Fig. 2:**
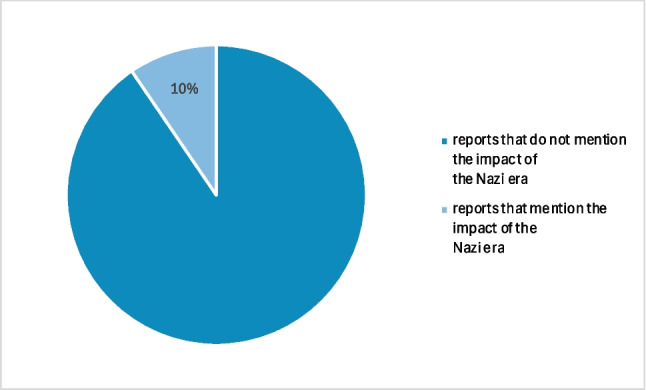
Proportion of reports relating to the Nazi era (reports on the war period) (source: Philippu, A (2004): Geschichte und Wirken der pharmakologischen, klinisch-pharmakologischen und toxikologischen Institute im deutschsprachigen Raum, Band 1)

It is noticeable that the National Socialist period is often avoided in literature, especially in biographies. This hypothesis is supported by the reports on the history of pharmacological, clinical-pharmacological, and toxicological institutes in German-speaking countries published by Athineos Philippu in his book “Geschichte und Wirken der pharmakologischen, klinisch-pharmakologischen und toxikologischen Institute im deutschsprachigen Raum” in 2004. (cf. Table 1). The Philippu presentation includes 33 relevant institutes. Of these, only three reported on the period of National Socialism and the consequences for the respective institute. This corresponds to 9% (cf. Fig. 1). Conversely, the period of National Socialism is completely omitted from the reports of the other 30 institutes. Developments during the Second World War are at least briefly documented in 19 of the 33 institute reports. Of these 19 institutes, however, only two report on the period of National Socialism or its consequences for the respective institute, which corresponds to approx. 10% (cf. Fig. 2). The deliberate destruction of documents could also play a role here.

**Table 1:**
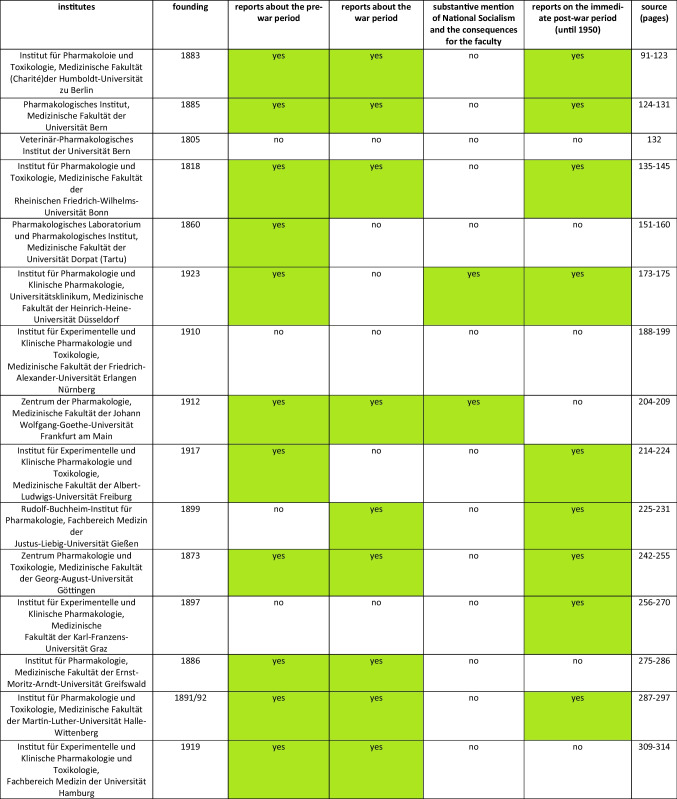

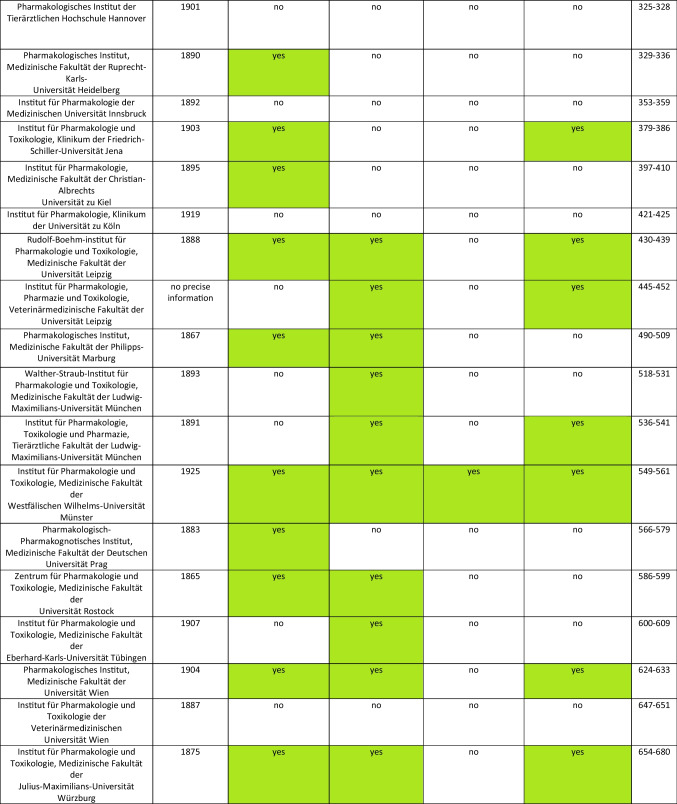
Overview of the German-speaking universities that existed during the Nazi era (source: Philippu, A (2004): Geschichte und Wirken der pharmakologischen, klinisch-pharmakologischen und toxikologischen Institute im deutschsprachigen Raum, Band 1)

Marked green: to particularly highlight yes statements

### DGP publications in Naunyn–Schmiedeberg Archive for Experimental Pathology and Pharmacology 1920–1950

In the years 1920–1950, the DGP board published a total of 45 articles in the Naunyn–Schmiedeberg Archiv für experimentelle Pathologie und Pharmakologie (now Naunyn Schmiedeberg’s Archives of Pharmacology). In terms of content, these are mainly lists of members, minutes of the business sessions of the general meetings, and announcements of the statutes. There are also some opening speeches from DGP conferences (cf. Table 2).

**Table 2:** Publications about annual meetings, speeches, and membership lists of the DGP in Naunyn–Schmiedeberg Archiv für experimentelle Pathologie und Pharmakologie

Original title	English title	Content	Date of publication	Volume	Pages
Deutsche Pharmakologische Gesellschaft	German Pharmacolocgical Society	Announcement of the founding of the German Pharmacological Society	01 September 1920	88	371
Verhandlungen der Deutschen pharmakologischen Gesellschaft—3. Tagung vom 20.–22.September 1922 in Leipzig	Negotiations of the German Pharmacological Society −3rd Meeting from September 20th–22nd, 1922 in Leipzig	Report on the third conference in Leipzig	01 November 1923	96	1–46
Mitteilung	Notice	Announcement of the next meeting at the end of September 1925 in Düsseldorf	01 September 1925	110	386
Mitgliederliste der Deutschen Pharmakologischen Gesellschaft	List of Members of the German Pharmacological Society	List of members as of 1924 > > total: 142	01 December 1925	105	23–26
Protokoll der Mitgliederversammlung der 6. Tagung der Deutschen Pharmakologischen Gesellschaft	Minutes of the General Meeting of the 6th Meeting of the German Pharmacological Society	Agenda: business matters, place and time of the next meeting, changes to the statutes, elections	01 September 1927	119	14–15
Vorstand 1926/28	Board of Directors 1926/28	Business: name of the board 1926, list of members	01 September 1927	119	5
Mitgliederliste der Deutschen Pharmakologischen Gesellschaft	List of Members of the German Pharmacological Society	> > total: 191, including 1 honorary member	01 September 1927	119	5–10
Satzungen der Deutschen Pharmakologischen Gesellschaft	Statutes of the German Pharmacological Society	Announcement of the statutes	01 September 1927	119	11–13
Geschäftliches	Business	Business: name of the board 1927, list of members	01 January 1928	128	6
Mitgliederliste der Deutschen Pharmakologischen Gesellschaft	List of Members of the German Pharmacological Society	> > total: 217, including 2 honorary members	01 January 1928	128	6–12
Satzungen der Deutschen Pharmakologischen Gesellschaft	Statutes of the German Pharmacological Society	Announcement of the statutes	01 January 1928	128	13–15
Geschäftliches	Business	Business meeting: agenda: business, elections, time and place of the next meeting, honorary memberships	01 January 1928	128	16
Geschäftliches	Business	Business: name of the board 1928, list of members	01 December 1928	138	6
Mitgliederliste der Deutschen Pharmakologischen Gesellschaft	List of Members of the German Pharmacological Society	> > total: 236, including 2 honorary members	01 December 1928	138	6–13
Geschäftssitzung	Business meeting	Agenda: business, elections, election of an honorary member, time and place of the next meeting, miscellaneous	01 December 1928	138	14
Vorstand 1929/30	Board of Directors 1929/30	Business: name of the board 1929, list of members	01 December 1929	147	5
Mitgliederliste der Deutschen Pharmakologischen Gesellschaft	List of Members of the German Pharmacological Society	> > total: 249, including 2 honorary members	01 December 1929	147	5–12
Geschäftssitzung	Business meeting	Agenda: business, election, time and place of the next meeting, miscellaneous	01 December 1929	147	13
Eröffnungsansprache	Opening speech	Opening address, author and location of the conference unknown	01 December 1929	147	14–17
Geschäftliches	Business	Business: name of the board 1930, list of members	01 December 1930	157	5
Mitgliederliste der Deutschen Pharmakologischen Gesellschaft	List of Members of the German Pharmacological Society	> > total: 257, including 2 honorary members	01 December 1930	157	5–12
Satzungen der Deutschen Pharmakologischen Gesellschaft	Statutes of the German Pharmacological Society	Announcement of the statutes	01 December 1930	157	13–15
Geschäftssitzung	Business meeting	Agenda: business, time and place of the next meeting, election, miscellaneous	01 December 1930	157	16
Eröffnungsansprache	Opening speech	Opening address of the 10th conference, given by J. Schüller	01 December 1930	157	17–21
Geschäftliches	Business	Business: name of the board 1931, list of members	01 December 1932	167	5
Mitgliederliste der Deutschen Pharmakologischen Gesellschaft	List of Members of the German Pharmacological Society	> > total: 260, including 2 honorary members	01 December 1932	167	5–12
Satzungen der Deutschen Pharmakologischen Gesellschaft	Statutes of the German Pharmacological Society	Announcement of the statutes	01 December 1932	167	13–15
Geschäftssitzung	Business meeting	Agenda: business matters, new board elections, miscellaneous	01 December 1932	167	16
Eröffnungsansprache	Opening speech	Opening speech, given by O. Loewi, location unknown	01 December 1932	167	17–20
Geschäftliches	Business	Business: name of the board 1934, list of members	01 January 1936	181	5
Mitgliederliste der Deutschen Pharmakologischen Gesellschaft	List of members of the German Pharmacological Society	> > total: 259, including 4 honorary members	01 January 1936	181	5–11
Satzungen der Deutschen Pharmakologischen Gesellschaft	Statutes of the German Pharmacological Society	Announcement of the statutes	01 January 1936	181	12–14
Geschäftssitzung	Business meeting	Agenda: business matters, new board elections, miscellaneous	01 January 1936	181	15
Geschäftssitzung	Business meeting	No agenda, seems to have taken place unscheduled, adoption of changes to the statutes that affect all clubs	01 January 1936	181	16
Eröffnungsansprachen	Opening speeches	Opening speeches, given by S. Janssen and W. Straub	01 January 1936	181	17–23
Mitgliederverzeichnis der Deutschen Pharmakologischen Gesellschaft	List of members of the German Pharmacological Society	Business: list of members 1935 > > total: 266, including 4 honorary members	01 January 1936	184	5–11
Satzungen der Deutschen Pharmakologischen Gesellschaft	Statutes of the German Pharmacological Society	Announcement of the statutes	01 January 1936	184	12–14
Geschäftssitzung	Business meeting	Agenda: business matters, time and place of the next meeting, election of honorary members, miscellaneous	01 January 1936	184	15
Eröffnungsansprachen	Opening speeches	Opening speeches, given by S. Janssen and F. Hildebrandt	01 January 1936	184	16–22
Mitgliederverzeichnis der Deutschen Pharmakologischen Gesellschaft	List of members of the German Pharmacological Society	Business: list of members 1936/38 > > total: 259, including 4 honorary members, statutes and business meeting	01 January 1938	190	6–16
Eröffnungsansprachen	Opening speeches	Opening speeches, given by F. Flury and W. Heubner	01 January 1938	190	17–29
A. Eröffnungsansprache	A. Opening speech	Opening speeches, given by B. Behrens und O. Riesser	01 January 1949	208	1–3
D. Schlußwort	D. Conclusion	Final word, given by O. Riesser, 15th meeting	01 January 1949	208	50
Zur Tagung der Deutschen Pharmakologischen Gesellschaft—Düsseldorf, am 11.9.1948—Eröffnungsrede	At the conference of the German Pharmacological Society—Düsseldorf, on September 11, 1948—Opening speech	Opening speech, given by O. Riesser, meeting 1948 in Düsseldorf	01 January 1949	208	51–57
A. Eröffnungsrede zur 17. Tagung der Deutschen Pharmakologischen Gesellschaft—in Bad Nauheim am 12. April 1950	A. Opening speech at the 17th meeting of the German Pharmacological Society—in Bad Nauheim on April 12, 1950	Opening speech of the 17th conference (1950), given by W. Heubner	01 January 1950	212	1–8

The membership lists and reports of the business meetings for the years 1924–1931, with the exception of 1925, are available in full. A steady growth in the number of members can be observed. In 1924, the DGP had 142 members, which had almost doubled to 260 by 1931. By 1938, the number of members had stagnated at around 260.

It is striking that during the Second World War and also in the immediate post-war period, there were no publications by the DGP Board. The last publications before the outbreak of the war are the list of members for 1938 and the opening addresses by Ferdinand Flury and Wolfgang Heubner. The reason for this could be that no further meetings were held after 1938 due to the outbreak of the Second World War, which also affected the general meetings, etc. (cf. Table 2).

The first publications after the end of the Second World War are from 1949, but these are only the opening speeches of the first meetings in the post-war period. Minutes of business meetings or membership lists were no longer published in 1949 and 1950 (cf. Table 2).

### Analysis of the first chairmen and managing directors before and after the Second World War

Due to the lack of data on board minutes, etc., from the period of National Socialism, the activities of the post-war board of the DGP were analyzed on the basis of the selection of its chairmen on National Socialism before the Second World War and immediately after the end of the war. These were the first chairman during the war, Behrend Walter Behrens (1895–1969), the first chairman after the end of the war, Otto Riesser (1882–1949), and the managing director after the end of the war, Hellmut Weese (1897–1954).

### Behrend Walter Behrens

**Fig. 3:**
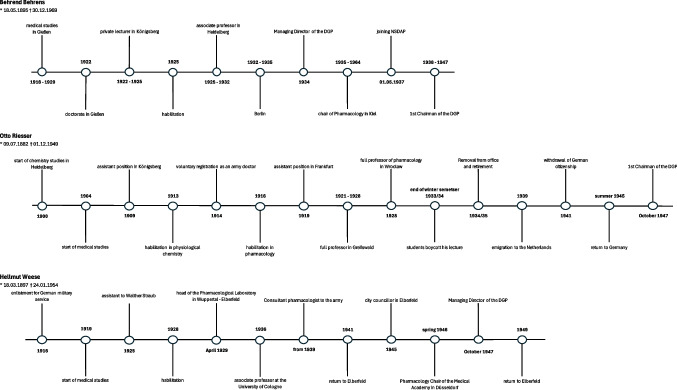

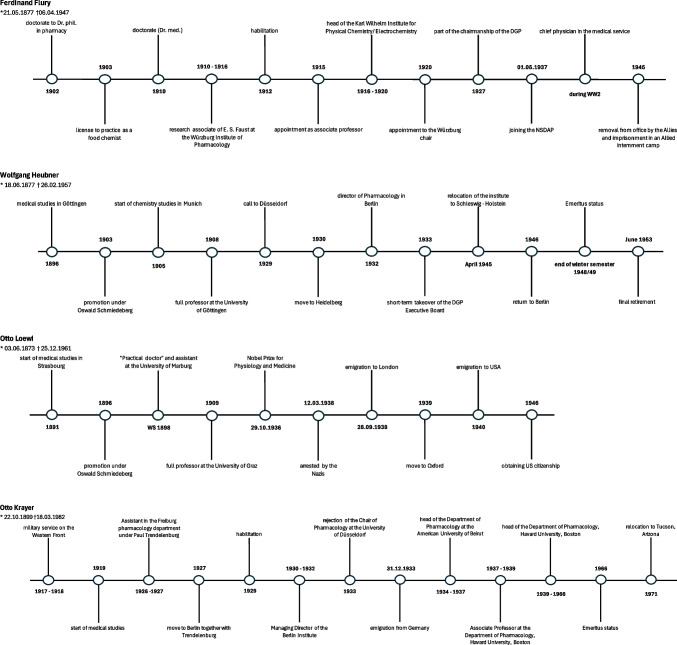
Biographical timeline of Behrend Behrens, Otto Riesser, Hellmut Weese, Ferdinand Flury, Wolfgang Heubner, Otto Loewi, and Otto Krayer

Behrend Walter Behrens was born on May 23, 1895, in Giessen. He pursued his medical studies at the University of Giessen from 1916 to 1920 and was awarded his doctoral degree in 1922. Before Behrens habilitated in Königsberg in 1925, he worked there as a research assistant from 1922 to 1925. In 1933, Behrens joined the SA and also became a member of the NSDAP on May 1, 1937. In 1934, Behrens was appointed Managing Director of the German Pharmacological Society (DGP). He was later elected as First Chairman in 1938, during the society’s final board meeting before the outbreak of the Second World War. He held this position until the society was re-established in 1947 (Lindner 1996; Kieler Gelehrtenverzeichnis n.d.). In 1934, Behrens succeeded Hermann Fühner (1871–1944) as editor of the collection of poisoning cases (now Archiv für Toxikologie). He was involved in rebuilding the journal after the end of the war until his retirement in 1966 (*Behrend *Behrens 1970). In 1935, Behrens took over the pharmacology chair at the University of Kiel but was also active in the Reichsministerium für Wissenschaft, Erziehung und Volksbildung (Reich Ministery of Science, Education and Culture). In 1942, Behrens and his assistants Gerhard Orzechowski (1902–1977) and Günther Malorny (1912–1978) were commissioned by the High Command of the Navy to carry out investigations in the field of military toxicology and combat medicine. Among other topics, they were to investigate the ventilation of small motor vehicles and submarines, the problems of keeping awake and carbonic acid poisoning. For this purpose, a pharmacological-toxicological laboratory was set up at the Kiel Pharmacological Institute, which belonged to the Research Institute for Submarine Medicine of the Navy. Due to his activities in the Reichwissenschaftsministerium in 1935/36, Behrens was briefly dismissed by the Allies from December 1945 to October 1946 (Ratschko 2018). Behrens retained the chair in Kiel until 1964. He died in Fallingbostel on December 30, 1969 (Lindner 1996; Fig. 3).

Between 1924 and 1959, Behrend Behrens published a total of 21 papers in the “Archiv für experimentelle Pathologie und Pharmakologie” (now Naunyn Schmiedeberg’s Archives of Pharmacology). His main topics were the pharmacology of lead and the effect of digitalis on the frog preparation (cf. Table 3).

**Table 3:** Behrend Behrens´ publications in Archiv für experimentelle Pathologie und Pharmakologie

Date	Theme German	Theme English	Authors	Volume	Pages
01 January 1924	Untersuchungen über den Mechanismus der Kochsalzvergiftungen—I. Mitteilung: Die Bedeutung örtlicher osmotischer Vorgänge	Studies into the mechanism of salt poisoning—I. message: the importance of local osmotic processes	**Behrend Behrens**	103	39–51
01 September 1925	Untersuchungen über Aufnahme, Ausscheidung und Verteilung kleinster Bleimengen	Investigations into the absorption, excretation and distribution of small amounts of lead	**Behrend Behrens**	109	332–357
01 December 1925	Resorption und Ausscheidung des Bleis	Absorption and excretation of lead	**Behrend Behrens**	105	6
01 September 1927	Zur Pharmakologie des Bleis—II. Mitteilung: Die Verteilung und der Zustand kleinster Bleimengen im Blut	On the pharmacology of lead—II. message: The distribution and status of small amounts of lead in the blood	**Behrend Behrens**, Reinhold Pachur	122	319–337
01 January 1928	Die Kochsalzazidose	Saline acidosis	**Behrend Behrens**	128	104–105
01 December 1928	Ein Gerät zur laufenden Messung des Atemvolumens beim Tier	A device for continuously measuring the respiratory volume of an animal	**Behrend Behrens**	138	166–167
01 December 1928	Zur Pharmakologie des Bleis—Mitteilung: Die Verteilung des Bleis zwischen Blut und Gewebe nach intravenöser Einspritzung	On the pharmacology of lead—message: The distribution of lead between blood and tissue	**Behrend Behrens**, Günther Anton	137	305–310
01 December 1928	Zur Pharmakologie des Bleis—Mitteilung: Über den Mechanismus der Bleivergiftung von Fischen	On the pharmacology of lead—message: On the mechanism of lead poisoning of fish	**Behrend Behrens**	137	311–314
01 March 1929	Zur Auswertung der Digitalisblätter im Froschversuch	For the evaluation of the digitalis leaves in the frog experiment	**Behrend Behrens**	140	237–256
01 May 1929	Spricht die Höhe des Blutdrucks kleiner Säugetiere gegen die Annahme eines Filtrationsprozesses in der Niere?—Experimentelle Ermittlung des Blutdrucks der weißen Maus	Does the high blood pressure of small mammals contradict the assumption of a filtration process in the kidneys?—Experimental determination of the blood pressure of the white mouse	**Behrend Behrens**	139	154–158
01 December 1932	Zur Pharmakologie des Bleis—VI. Mitteilung: Eine Methode zur Bestimmung kleiner Bleimengen	On the pharmacology of lead—VI. message: A method for determining small amounts of lead	**Behrend Behrens**, Hans Ommo Behrens	164	501–508
01 December 1932	Auswertung von Digitalispräparaten des Handels und von Apothekenzubereitung	Evaluation of commercial digitalis preparations and pharmacy preparations	**B. Behrens**, Gros, Hildebrandt	167	365–380
01 December 1933	Zur Pharmakologie der Metaphosphorsäuren	On the pharmacology of metaphosphoric acids	**Behrend Behrens**, Karl Seelkopf	169	238–245
01 March 1934	Wie sind Reihenversuche für biologische Auswertungen am zweckmäßigsten anzuordnen?	What is the most expedient way to arrange serial experiments for biological evaluations?	**B. Behrens**, G. Kärber	177	379–388
01 January 1935	Vergleichende Untersuchungen über die Wirksamkeit von Adrenalin und p-I-Sympatol	Comparative studies on the effectiveness of adrenaline and p-I-Sympatol	**B. Behrens**, H. Taeger	178	64–85
01 March 1937	Über Auswertung von Follikelhormon-Präparaten	About evaluation of follicular hormone preparations	W. Heubner, W. Koll, F. Külz	186	121–160
01 March 1939	Brechenerregende Substanzen im Kaffee und ihre Bedeutung für die Unverträglichkeit des Kaffeegetränkes	Vomiting substances in coffee and their significance for intolerance to the coffee drink	**Behrend Behrens**, Günther Malorny	194	369–388
01 July 1949	Eine Methode der fortlaufenden Blutdruckregistrierung an freigelegten, nicht eröffneten Arterien	A method of continuous blood pressure recording on exposed, unopened arteries	**Behrend Behrens**, Günter W. Jacobi	206	450–458
01 May 1952	Ein Direktschreibersystem zur Blutdruckregistrierung am uneröffneten Gefäß	A direct recorder system for recording blood pressure on an unopened vessel	**B. Behrens**, G.W. Jacobi	215	409–412
01 January 1957	Beitrag zur Bestimmung der LD50 und der Berechnung ihrer Fehlerbreite	Contribution to the determination of the LD50 and the calculation of its error range	**Behrend Behrens**, Lucie Schlosser	230	59–72
01 January 1959	Apparatur zur laufenden Messung von Atemvolumen und Atemfrequenz bei kleinen Versuchstieren (Kaninchen)	Apparatus for the continuous measurement of respiratory volume and respiratory frequency in small laboratory animals (rabbits)	**B. Behrens**, L. Schlosser	236	301–303

### Otto Riesser

Otto Riesser was born on July 9, 1882, in Frankfurt am Main in a German-Jewish family. His father Jacob Riesser (1853–1932) converted to the Protestant faith together with his three children. Only Riessers’ mother, Emilie Riesser (1858–1945), remained true to the Jewish faith. In 1895, Otto Riesser was baptized protestant. After completing his chemistry degree in Heidelberg in 1903, he enrolled in the Faculty of Medicine, as his interest in physiology had been awakened, and studied until 1908. Due to severe hearing loss, Riesser had to interrupt his medical studies until he received one of the new telephone hearing aids developed in the USA. In 1909, Riesser accepted an assistant position with Max Jaffé in Königsberg. With the outbreak of the First World War in 1914, he volunteered to serve as a doctor at the front, which is why the Ministry of the Interior granted him a license to practice medicine without an examination, which was a special provision. In 1928, Riesser succeeded Julius Pohl as full professor of pharmacology in Breslau. Professionally speaking, the years in Breslau were the high point of his career as a university lecturer, but he never managed to settle in completely in Breslau. At the end of the 1933/34 winter semester, students boycotted his lectures for the first time by staying away. Although Riesser, himself, a member of a student fraternity, initially welcomed the National Socialist takeover in 1933, he later became a source of concern for the regime due to his growing disassociation from National Socialist ideology. As a result, he was removed from office in 1934 on racial grounds and was forcibly stripped of his academic title in 1935 (Philippu 2011, 2014; Hock 2024). After his brief arrest in the course of the Reichspogromnacht of November 9–10, 1938, Riesser fled to the Netherlands in 1939, where he was able to work until 1941 at the pharmacological institute in Amsterdam with Ernst Laqueur (1880–1947). Due to his Jewish origin, he was dismissed from the pharmacological institute in October 1941. He was also stripped of his German citizenship in December 1941, which meant he also lost his pension. Without his pension, Riesser was no longer able to support his wife and children in Germany. After his dismissal, Riesser was still able to make a living in a small private laboratory by taking on contracts from industry. In the fall of 1944, he had to stop working for good due to a lack of gas and electricity. His marriage to an “Aryan” woman, his German nationalist convictions, and a large number of coincidences initially offered him a  certain degree of protection from deportation, which he managed to evade until the end of the regime (Löffelholz and Trendelenburg 2008; Hock 2024). Riesser returned to Germany in August 1945. Due to the desolate conditions in Germany, Riesser saw no opportunities for academic work, which is why he accepted the position of special advisor at the Greater Hessian State Ministry for Culture and Education in December 1945. In 1947, he was elected First Chairman of the DGP. His chairmanship ended with his sudden death on December 1, 1949, in Frankfurt am Main (Hock 2024; Löffelholz and Trendelenburg 2008; Protokoll der Vorstandssitzung of October 7, 1947, ArchMHH, Dep. 13, 2; Fig. 3).

Between 1916 and 1950, Otto Riesser published a total of 41 papers in the “Archiv für experimentelle Pathologie und Pharmakologie” (now Naunyn Schmiedeberg’s Archives of Pharmacology). He mainly dealt with the physiology of striated muscle (cf. Table 4).

**Table 4:** Otto Riessers publications in Archiv für experimentelle Pathologie und Pharmakologie

Date	Theme German	Theme English	Authors	Volume	Pages
01 September 1916	Über Tonus und Kreatingehalt der Muskeln in ihren Beziehungen zu Wärmeregulation und zentral-sympathischer Erregung	About the tone and creatine content of the muscles in their relationship to heat regulation and central sympathetic excitation	**Otto Riesser**	80	183–230
01 November 1921	Physiologische und kolloidchemische Untersuchungen über den Mechanismus der durch Gifte bewirkten Kontraktur quergestreifter Muskeln	Physiological and colloid-chemical studies on the mechanism of contracture of striated muscles caused by poisons	**Otto Riesser**, S.M. Neuschloß	91	342–365
01 May 1922	Physiologische und kolloidchemische Untersuchungen über den Mechanismus der durch Gifte bewirkten Kontraktur quergestreifter Muskeln	Physiological and colloid-chemical studies on the mechanism of contracture of striated muscles caused by poisons	**Otto Riesser**, S.M. Neuschloß	94	190–221
01 July 1922	Physiologische und kolloidchemische Untersuchungen über den Mechanismus der durch Gifte bewirkten Kontraktur quergestreifter Muskeln—Über die durch Nikotin und Kaliumsalze ausgelöste Erregungskontraktur des Froschmuskels und über die rezeptive Substanz Landleys	Physiological and colloid-chemical studies on the mechanism of the contracture of striated muscles caused by poisons—On the excitation contracture of the frog muscle triggered by nicotine and potassium salts and on Landley’s receptive substance	**Otto Riesser**, S.M. Neuschloß	92	254–272
01 July 1922	Physiologische und kolloidchemische Untersuchungen über den Mechanismus der durch Gifte bewirkten Kontraktur quergestreifter Muskeln—Über die durch Nikotin und Kaliumsalze ausgelöste Erregungskontraktur des Froschmuskels und über die rezeptive Substanz	Physiological and colloid-chemical studies on the mechanism of the contracture of striated muscles caused by poisons—On the excitation contracture of the frog muscle triggered by nicotine and potassium salts and on the receptive substance	**Otto Riesser**, S.M. Neuschloß	92	254–272
01 July 1922	Berichtigung	Correction	**Otto Riesser**	92	393
01 July 1922	Physiologische und kolloidchemische Untersuchungen über den Mechanismus der durch Gifte bewirkten Kontraktur quergestreifter Muskeln—III. Über den Mechanismus der Coffeinkontraktur	Physiological and colloid-chemical studies on the mechanism of contracture of striated muscles caused by poisons—III. On the mechanism of caffeine contracture	**Otto Riesser**, S.M. Neuschloß	93	163–178
01 September 1923	Über die Muskelwirkung des Kampfers; nach Versuchen am isolierten Froschgastrocnemius	On the muscular effects of camphor; after experiments on isolated frog gastrocnemius	Olga Tschernewa, **Otto Riesser**	99	346–364
01 January 1927	Untersuchungen über die elektrische Reizung des überlebenden Kaninchendünndarmes	Studies on the electrical stimulation of the surviving rabbit small intestine	Friedrich Pels Leusden, **Otto Riesser**	120	77–99
01 September 1927	Vergleichend pharmakologische Untersuchungen an Muskeln von Avertebraten	Comparative pharmacological studies on muscles of avertebrates	**Otto Riesser**	120	282–313
01 January 1928	Fortgesetzte vergleichend pharmakologische und physiologische Untersuchungen an den Muskeln von Meerestieren	Continued comparative pharmacological and physiological studies on the muscles of Sea creatures	**Otto Riesser**, Anneliese Hansen	134	1–16
01 December 1928	Über die Milchsäurebildung des freischlagenden Froschherzens—nach Versuchen von Dr. Nagaya	About the formation of lactic acid in the free-beating frog heart—according to experiments by Dr. Nagaya	**Riesser**	138	136–137
01 December 1929	Azetaldehyd aus Eiweiß	Acetaldehyde from protein	**Riesser**	147	85
01 January 1930	Untersuchungen über die Entstehung der Kreatinurie—Mitteilung: Azidose und Kreatinurie	Studies on the development of creatinuria—message: Acidosis and creatinuria	**Otto Riesser**, Carlo Brentano	155	1–20
01 May 1930	Über die Wirkung von Bestrahlung auf die Giftempfindlichkeit weißer Mäuse	On the effect of irradiation on the sensitivity of white mice to poison	**Otto Riesser**, Alfons Hadrossek	155	139–159
01 December 1930	Untersuchungen am Musculus rectus abdominis des Frosches—I. Mitteilung: Die “passive Verkürzungsreaktion” und ihre Bedeutung für die Theorie der tonischen Kontraktur	Investigations on the rectus abdominis muscle of the frog—I. message: The “passive shortening reaction” and its significance for the theory of tonic contracture	**Otto Riesser**, Tatsunori Masayama	156	26–36
01 July 1931	Beiträge zur Kenntnis des Azetylcholins	Contributions to the knowledge of acetylcholine	**Otto Riesser**	161	34–58
01 December 1933	Über die Zusammensetzung einiger Kombinationspräparate (Antineuralgika) des Handels	About the composition of some combination preparations (antineuralgics) on the market	**Otto Riesser**	169	164–179
01 December 1933	Über die Beeinflussung des Muskelchemismus durch intravenöse Infusion von vegetativen Giften und Narkotika	On the influence of muscle chemistry by intravenous infusion of vegetative poisons and narcotics	**Otto Riesser**, Keniti Yamada	170	208–225
01 December 1933	Die passive Verkürzbarkeit der Muskeln als Kennzeichen tonischer Verkürzungsreaktionen	The passive shortening ability of the muscles as a characteristic of tonic shortening reactions	**Otto Riesser**, Oswald Hansel	170	571–579
01 December 1933	Pharmakologische Untersuchungen am Musculus rectus des Frosches	Pharmacological studies on the rectus muscle of the frog	**Otto Riesser,** Philipp Klein	170	580–591
01 December 1933	Fortgesetze pharmakologische Untersuchungen an den Muskeln wirbelloser Meerestiere	Continued pharmacological studies on the muscles of marine invertebrates	**Otto Riesser**	172	194–212
01 January 1934	Über Resorption und Speicherung verfütterten Calciums bei Kaninchen	About absorption and storage of fed calcium in rabbits	**Otto Riesser**, Kurt Salomon, Lucie Karbe	175	38–61
01 March 1934	Zur Frage des Einflusses von Digitoxin und Strophanthin auf oxydative Vorgänge in Versuchen am Modell sowie am atmenden überlebenden Herzmuskelgewebe	On the question of the influence of digitoxin and strophanthin on oxidative processes in experiments on models and on breathing surviving cardiac muscle tissue	Kurt Salomon, **Otto Riesser**	177	450–462
01 March 1934	Über die Abhängigkeit der narkoticawirkung am Muskel von der Calciumkonzentration und die Bedeutung des Calciums für die Erregbarkeit der motorischen Nervenendigungen	On the dependence of the narcotic effect on the muscle on the calcium concentration and the importance of calcium for the excitability of the motor nerve endings	Hans Schein, **Otto Riesser**	177	463–474
01 March 1935	Über Calciummobilisierung durch Salze der Brenzcatechindisulfosäure	About calcium mobilization by salts of catechol disulfonic acid	**Otto Riesser**, Lucy Karbe	178	455–460
01 November 1935	Zur Methodik der Bestimmung der Blutgerinnungs-Geschwindigkeit	On the methodology for determining blood clotting speed	**Otto Riesser**, Alfred Nagel	179	743–747
01 November 1935	Über die gerinnungsförderde Wirkung saurer Substanzen, insbesondere des Pektins	About the coagulation-promoting effect of acidic substances, especially pectin	**Otto Riesser**, Alfred Nagel	179	748–760
01 January 1937	Histaminstudien	Histamine studies	**Otto Riesser**	187	1–21
01 March 1938	Zur Methodik vergleichender Bestimmung zentraler Erregungswirkungen; Kaffee-Versuche	On the methodology of comparative determination of central arousal effects; Coffee trials	**Otto Riesser**	189	151–156
01 March 1942	Über die Wirkung der Ernährungsart auf die Insulin-Schockempfindlichkeit normaler und hypophysenloser Ratten und über den Einfluß des Ernährungszustands auf die Aktivität der innersekretorischen Drüsen	On the effect of the type of diet on the insulin shock sensitivity of normal and pituitary rats and on the influence of the nutritional status on the activity of the internal secretory glands	**Otto Riesser**	199	196–215
01 March 1948	Betrachtungen über das Acetylcholin und die Übertragung der Erregung vom Nerven auf den Muskel	Considerations about acetylcholine and the transmission of excitation from the nerve to the muscle	**Otto Riesser**	205	340–350
01 January 1949	Der gegenseitige Antagonismus von Insulin und Adrenalin-Adrenochrom bei der Glykogenbildung im isolierten Rattenzwerchfell—II. Mitteilung	The mutual antagonism of insulin and adrenaline-adrenochrome in glycogen formation in the isolated Rat diaphragm—II. message	**Otto Riesser**, Horst Weeke, Leopold Ther	206	12–23
01 January 1949	A. Eröffnungsansprache	A. Opening remarks	**Otto Riesser**	208	3
01 January 1949	Pharmakologische Versuche über die Beeinflussug der Glykogensynthese im überlebenden Rattenzwerchfell	Pharmacological experiments on the influence of glycogen synthesis in the surviving rat diaphragm	**O. Riesser**, H. Weeke	208	19
01 January 1949	D. Schlußwort	D. Conclusion	**Otto Riesser**	208	50
01 January 1949	zur Tagung der Deutschen Pharmakologischen Gesellschaft	to the conference of the German Pharmacological Society	**Otto Riesser**	208	51–57
01 January 1949	Muskelpharmakologie und Klinik	Muscle pharmacology and clinic	**O. Riesser, H. Weeke**	208	133–144
01 March 1949	Ernst Laqueur, geb. 7.8.1880, gest. 10.8.1947	Ernst Laqueur, born August 7, 1880, died August 10, 1947	**Otto Riesser**	206	117–123
01 January 1950	Über den Einfluß des Diäthylaminoäthanols auf die Wirkungen von Adrenalin, Arterenol und Acetylcholin am isolierten Froschherz	On the influence of diethylaminoethanol on the effects of adrenaline, arterenol and acetylcholine on the isolated frog heart	**Otto Riesser**, Josef Hergott	209	95–103
01 January 1950	Einfluß der Wasserstoffionenkonzentration auf den gegenseitigen Antagonismus von Adrenalin-Adrenochrom und Insulin im Gykogensyntheseversuch am überlebenden isolierten Rattenzwerchfell	Influence of hydrogen ion concentration on the mutual antagonism of adrenaline-adrenochrome and insulin in the glycogen synthesis experiment on the surviving isolated rat diaphragm	**Otto Riesser,** Hansgünter Kahlfeld	210	437–443

### Hellmut Weese

Hellmut Weese was born in Munich on March 18, 1897. He studied medicine in Bern, Zurich, and Munich from 1919, graduating in 1924. From October 1925, Weese worked as a second assistant to Walther Straub. In April 1929, he took over the management of the Pharmacological Laboratory of I.G. Farbenindustrie AG in Wuppertal-Elberfeld until he accepted the position of associate professor at the University of Cologne in 1936. In 1939, Weese was appointed to the Wehrmacht as a consultant pharmacologist. He took part in the French, Greek, and Russian campaigns until the winter of 1941. During this time, Weese worked with his Elberfeld laboratory on a “well-tolerated blood replacement fluid” that was adapted to the conditions at the front. He was sent back to Elberfeld on “working leave,” where his research included the “prevention of adhesions after operations and wounds.” Despite this, Weese was considered to be untainted after the Second World War and was appointed to the Chair of Pharmacology at the Düsseldorf Medical Academy in 1946. At the same time, however, he was allowed to continue as head of the Pharmacological Institute of Bayer AG in Elberfeld, where he returned in 1949. In 1947, the pharmacologist was elected managing director and deputy chairman of the DGP. Weese was not only involved in the reconstitution of the DGP but also carried out important preparatory work for the founding of the DGAI in 1953. Weese died on January 24, 1954, as a result of an accident in his laboratory in Elberfeld (Krayer 1998, Philippu 2021; BArch R 9361-II/1026697 n.d.; Fig. 3).

Between 1926 and 1954, Hellmut Weese published a total of 19 papers in the “Archiv für experimentelle Pathologie und Pharmakologie” (now Naunyn Schmiedeberg’s Archives of Pharmacology). His research focused primarily on the effects of digitalis preparations (cf. Table 5).

**Table 5:** Hellmut Weese’s publications in Archiv für experimentelle Pathologie und Pharmakologie

Date	Theme German	Theme English	Authors	Volume	Pages
01 May 1926	Über die uteruswirksamen Substanzen im Mutterkorn- II. Teil: Histamin	About the substances in the ergot that have an effect on the uterus—Part II: Histamine	A.W. Forst, **H. Weese**	117	232–239
01 January 1928	Über die Beeinflussung der Rattenrachitis durch gelben Phosphor	On the influence of yellow phosphorus on rat rickets	**H. Weese**	135	111–117
01 May 1928	Über eine einfache Beatmungspumpe mit aktivem In- und Exspirium	Via a simple ventilation pump with active inhalation and expiration	**H. Weese**	135	210–212
01 May 1928	Digitalisverbrauch und Digitaliswirkung im Warmblüter—I. Mitteilung: Die Effektivdosen verschiedener Digitalisglykoside für das Herz	Digitalis consumption and digitalis effects in warm-blooded animals—I. message: The effective doses of various digitalis glycosides for the heart	**H. Weese**	135	228–244
01 September 1928	Digitalisverbrauch und Digitaliswirkung im Warmblüter—Berichtigung	Digitalis consumption and digitalis effect in warm-blooded animals—correction	**H. Weese**	136	?
01 December 1928	Digitalisverbrauch und Digitaliswirkung im Warmblüter—Einzelvorträge	Digitalis consumption and digitalis effects in warm-blooded animals—individual lectures	**H. Weese**	138	133–134
01 December 1928	Eine einfache Beatmungspumpe mit aktivem In- und Exspirium—Vorweisungen	A simple ventilation pump with active inhalation and expiration—instructions	**H. Weese**	138	167
01 May 1929	Digitalisverbrauch und Digitaliswirkung im Warmblüter—II. Mittteilung: Der extrakardiale Digitalisverbrauch und die Bedingungen der Glykosidbindung am Herzen	Digitalis consumption and digitalis effects in warm-blooded animals—II. message: Extracardiac digitalis consumption and the conditions of glycoside binding in the heart	**H. Weese**	141	329–350
01 January 1930	Digitalisverbrauch und Digitaliswirkung im Warmblüter—III. Mitteilung: Zur Entstehung der Kumulation	Digitalis consumption and digitalis effects in warm-blooded animals—III. message: On the emergence of accumulation	**H. Weese**	150	14–20
01 December 1932	Eine mechanische, automatisch registrierende Stromuhr für den geschlossenen Kreislauf	A mechanical, automatically registering current clock for the closed circuit	**H. Weese**	166	392–394
01 December 1932	Der Strophanthinaufbrauch des Warmblüterherzens im Fieber	The strophanthin consumption of the warm-blooded heart in fever	**H. Weese**	168	223–227
01 December 1933	Zur biologischen Auswertung des Kallikreins	For the biological evaluation of kallikrein	**H. Weese**	173	36–41
01 March 1934	Zur Kumulation der Digitalisglykoside	For the accumulation of digitalis glycosides	**H. Weese**, J. Dieckhoff	176	274–282
01 July 1934	Grundsätzliches zur experimentellen Pharmakologie der Digitalis, zugleich eine Entgegnung auf die Arbeit von Haferkorn und Lendle	Basic information on the experimental pharmacology of digitalis, at the same time a response to the work of Haferkorn and Lendle	**H. Weese**	175	754–758
01 July 1936	Berichtigung	Correction	**H. Weese**	181	739
01 January 1949	Blutersatzprobleme	Blood replacement problems	**H. Weese**	208	5–6
01 January 1949	Die Wirkung der Digitalis beim herzkranken Menschen	The effect of digitalis in people with heart disease	E. Edens, **H. Weese**	208	6–18
01 January 1954	“Potenzierte Narkos” und “Hilbernation durch Phenothiazine”. Einführung ins Hauptthema	“Potentiated anesthesia” and “Helbernation by phenothiazines”. Introduction to the main topic	**H. Weese**	222	15–20
01 January 1954	Freie Diskussion zum Hauptthema 1	Free discussion on the main topic 1	**Weese**, R. Thauer, G. Malorny, J. Pichotka, Schneider, Petry, K. Dietmann, C. Schmidt, Berendt, Willems, Strohmayer, Aebert, v. Werz, Koss & Laborit	222	79–87

### Awarding of honorary memberships and Schmiedeberg Plaques

The following analysis examines the awards conferred by the German Pharmacological Society (DGP) between 1948 and 1980, with a particular focus on the extent to which individuals persecuted by the National Socialist regime for political or racial reasons were considered in the selection of honorees and honorary memberships. Using data from Schneider et al. (2025), it was also analyzed which honorary memberships or award recipients were members of a National Socialist organization.

According to the DGP statutes of June 15, 1957, § 5, honorary membership is awarded to pharmacologists who have made outstanding contributions to their field. Proposals for this can be made either by the Board or by the General Assembly and require a 4/5 majority in the General Assembly for acceptance. Honorary members have all the same rights as ordinary members but are exempt from paying membership fees (Satzungen der Deutschen Pharmakologischen Gesellschaft Sitz Düsseldorf e.V., June 15, 1957, ArchMHH, Dep. 13, 35).

A total of 41 honorary memberships were awarded in the years 1926–1980. Among them are eight pharmacologists of Jewish descent and two pharmacologists who emigrated for political reasons. It is striking that four of the awards were made immediately after the DGP was re-established. Ernst Peter Pick received this recognition in 1950, Otto Loewi in 1951, Otto Krayer in 1952, and Rolf Meier in 1953. On the other hand, nine pharmacologists who were proven to have belonged to a National Socialist organization were also honored (cf. Table 6).

**Table 6:**
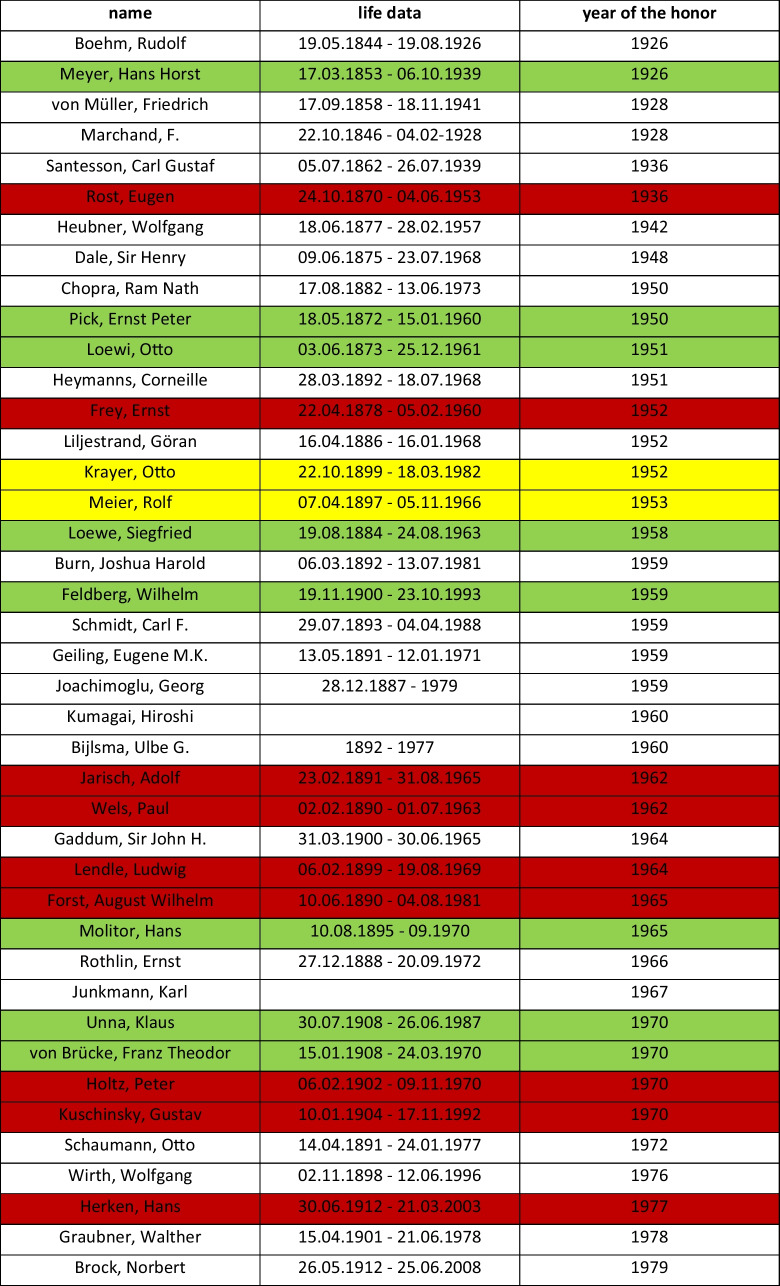
Honorary memberships of the DGPT until 1980

Marked green: member of the DGP who were  Jewish origin or Jewish ancestors, marked yellow: pharmacologists who rejected Nazi policies and therefore emigrated, marked red: member of the DGP who were members in a nazi-organisation (Schneider, H.; Wolters, C.; Seifert, R. (2025))

The most important honor of the DGP was and is the Schmiedeberg Plaque. According to the statutes of June 15, 1957, § 19, it is awarded to pharmacologists who have achieved merit in their field. This requires a unanimous proposal from the Executive Board, which is decided on by the General Assembly. To accept the proposal, a 2/3 majority vote of the general meeting is required (Satzungen der Deutschen Pharmakologischen Gesellschaft Sitz Düsseldorf e.V., June 15, 1957, ArchMHH, Dep. 13, 35).

A total of 25 Schmiedeberg plaques were awarded in the years 1956–1980. Among the award winners are seven pharmacologists who were racially persecuted during the National Socialist era due to their Jewish descent and had to emigrate, and two pharmacologists who did so for political reasons. In contrast, just two pharmacologists who were demonstrably members of a National Socialist organization were also honored here (cf. Table 7).

**Table 7:**
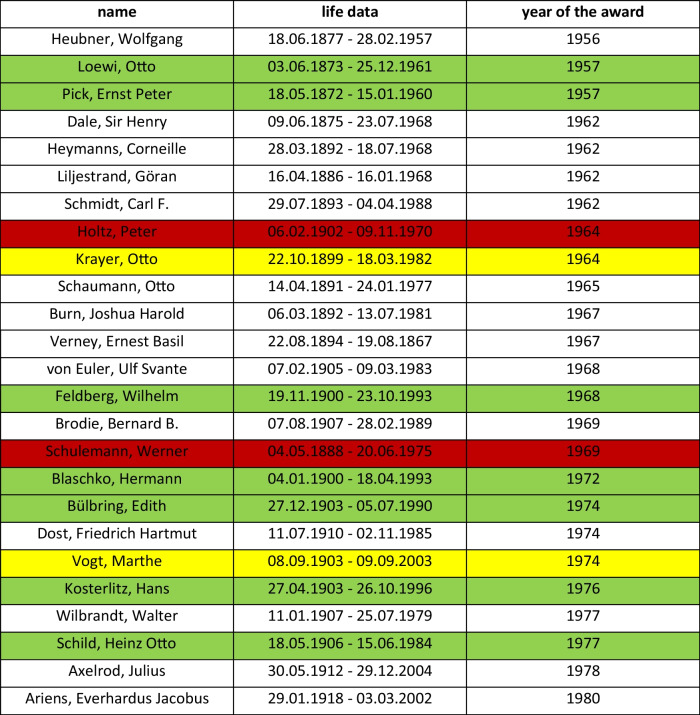
Schmiedeberg Plaques of the DGPT until 1980

Marked green: member of the DGP who were Jewish origin or Jewish ancestors, marked yellow: pharmacologists who rejected Nazi policies and therefore emigrated, marked red: member of the DGP who were members in a nazi-organisation (Schneider, H.; Wolters, C.; Seifert, R. (2025))

### Analysis of opening speeches at the 1938 and 1949 General Assemblies

Among the members of the DGP, as in many other institutions, there was no uniform position on National Socialist policies. Research in the DGP archives revealed four very interesting opening speeches, which reflect the different positions, both directly and indirectly addressed. The speeches were given by chairmen, honorary chairmen, or leading members of the DGP.

### 14th session from April 24–28, 1938, in Berlin

The two keynote speakers at the 14th conference, held in Berlin from April 24 to 28, 1938—just before the outbreak of the Second World War—were Ferdinand Flury and Wolfgang Heubner.

Ferdinand Flury was born in Würzburg on May 21, 1877. He first studied pharmacy in Erlangen and was awarded his doctorate in 1902. This was followed by additional studies in 1903, which Flury completed with a license to practice food chemistry and a degree in medicine. In 1910, Flury received his license to practice medicine and was awarded his doctorate. He worked at the Institute of Pharmacology at the University of Würzburg until 1916, where he habilitated in pharmacology in 1912. In 1915, Flury was appointed associate professor. In 1920, he was appointed to the Chair of Pharmacology at the University of Würzburg. As the first chairman of the DGP, Flury chaired the 7th conference in Würzburg in 1927. On May 1, 1937, Flury joined the NSDAP. In 1945, Flury was removed from office and arrested and taken to an Allied internment camp (Philippu 2004; Ferdinand Flury 2024; Fig. 3). Flury died in Würzburg on April 6, 1947 (Lindner 1996).

Wolfgang Heubner was born in Leipzig on June 18, 1877. After leaving school in 1896, he studied medicine in Göttingen, Berlin, and Strasbourg, where he was awarded his doctorate by Oswald Schmiedeberg in 1903. In 1905, Heubner began studying chemistry in Munich, later in Zurich, which he did not complete as Schmiedeberg made him his assistant. He then returned to Strasbourg. Via Göttingen, Düsseldorf, and Heidelberg, Heubner finally came to Berlin in 1932, where he worked as director of the Institute of Pharmacology. As director, Heubner himself had to dismiss civil servants from their jobs due to the “Gesetzes zur Wiederherstellung des Berufsbeamtentums” (“Law for the Restoration of the Professional Civil Service”). Heubner was never a member of the NSDAP but did not see himself as an “outspoken opponent of National Socialism,” as can be seen from his diaries. For example, Heubner supported several active National Socialists at his institute, above all Hermann Duckrey (1904–1994). It is also unclear whether Heubner was involved in the “seawater experiments” in the Dachau concentration camp, where he was called in as a consultant (Metzdorf 2022; Lindner 1996; Schagen 2008). In 1933, Heubner briefly took over the chairmanship of the DGP when the previous board had to dissolve (Muscholl 1995). Apart from a short leave of absence, during which Behrens briefly took over the chair as his senior assistant, Heubner remained in office in Berlin until 1945. In April 1945, he relocated the Berlin institute to Schleswig–Holstein due to the fighting around Berlin. Heubner was initially denied a return to Berlin by the British occupation authorities after the end of the war but succeeded in doing so at the beginning of 1946. In the post-war period, Heubner was heavily involved in the reconstruction of Germany’s universities and the resumption of teaching activities there. He held the chair at Berlin University, now reopened as Humboldt University, until 1949. From 1950 to 1952, he was the first professor of pharmacology at the newly founded Free University in West Berlin. Heubner died in Heidelberg on February 26, 1957 (Metzdorf 2022; Lindner 1996; Hoffmann et al. 2010; Fig. 3).

Flury began his opening speech by welcoming those present, in particular, the representatives of the Reich and state authorities, the party organizations, the Reich offices, representatives of the Wehrmacht, and many others. It is reported that Flury addressed particularly warm words “to the members from Austria, whom he could joyfully welcome for the first time as members of the German Reich” (Flury, Eröffnungsansprache, Verhandlungen der DGP (14) 1938, p. 17 n.d.). Only then does he also welcome the honorary members. Flury’s National Socialist attitude becomes clear at several points in the speech. The conclusion of the speech is particularly striking, in which he clearly declares his support for National Socialism: “Nicht zuletzt durch die Synthese von Laboratoriumsarbeit und Praxis hat sie [die Pharmakologie] gezeigt, daß sie eine lebenswichtige und lebensnahe Wissenschaft ist, die, wenn auch in der Stille, arbeitet und dabei Werte erhält und neue Werte schafft, im Dienste der Volksgesundheit, der Volkswirtschaft, des Volkswohls. So will auch die deutsche Pharmakologie beitragen, das deutsche Volk gesund, stark und arbeitsfähig zu erhalten. So reiht sie sich, ihrer Verpflichtung bewußt, auch als politischer Faktor ein in den Dienst unserer großen Gemeinschaft, in das Aufbauwerk des Führers. Dabei soll uns niemand übertreffen in ehrlichem Wollen, an Arbeitsfreudigkeit, an Lauterkeit der Gesinnung. Ehe wir nun zur Arbeit schreiten, gilt unsere Huldigung dem ersten Arbeiter unseres Volkes, dem Schirmherren der deutschen Wissenschaft, dem Schöpfer des neuen Großdeutschen Reiches. Wir grüßen in dankbarer Verehrung und treuer Ergebenheit unserer Führer Adolf Hitler mit einem dreifachen Sieg-Heil!” (“Not least through the synthesis of laboratory work and practice, it [pharmacology] has shown that it is a vital and life-oriented science that works, albeit in silence, and in doing so preserves values and creates new values in the service of public health, the national economy and the welfare of the people. German pharmacology also wants to contribute to keeping the German people healthy, strong and able to work. Thus, aware of its obligation, it also joins the ranks as a political factor in the service of our great community, in the Führer’s work of reconstruction. In this, no one should surpass us in honest will, in enthusiasm for work, in sincerity of spirit. Before we set to work, we pay homage to the first worker of our nation, the patron of German science, the creator of the new Greater German Reich. We greet our leader Adolf Hitler with grateful reverence and loyal devotion with a threefold Sieg-Heil!”) (Flury, Eröffnungsansprache, Verhandlungen der DGP (14) 1938, p. 24 n.d.).

Heubner’s speech follows in contrast to this. Unlike Flury, Heubner does not welcome political representatives but simply thanks some of the ministries involved in organizing the conference. In contrast to Flury’s ingratiating words, Heubner emphasizes: “Und niemand wird wohl ernstlich glauben, daß man etwa durch einen Befehl, durch den Appell an ein Pflichtgefühl, das Bemühen um theoretische Erkenntnis zu gleicher Anspannung bewegen könnte, wie es die innere Anziehungskraft des geistigen Interesses tut.” (“And no one will seriously believe that an order, an appeal to a sense of duty, could motivate efforts to gain theoretical knowledge to the same extent as the inner attraction of intellectual interest.”) (Heubner, Eröffnungsansprache, Verhandlungen der DGP (14) 1938, p. 27 n.d.), which can be interpreted as a rejection of the dictatorial approach of the National Socialists. In the further course of the speech, Heubner reveals his aversion to the racial ideology of the National Socialists. He emphasizes: “Diese Dankbarkeit ist wohl die eigentliche Wurzel der weltumspannenden Verbundenheit der Gelehrten, in der die Frage nach Herkunft oder Abkunft gleichgültig ist gegenüber der Frage nach dem Beitrag des Einzelnen zu der Beglückung des Geistes.” (“This gratitude is probably the real root of the global solidarity of scholars, in which the question of origin or descent is irrelevant to the question of the individual’s contribution to the happiness of the spirit.”) Furthermore, Heubner indirectly alludes to the arrest of Jewish member Otto Loewi with the words: “So werden viele Seelen davon berührt, wenn Unglück hereinbricht über einen hervorragenden Entdecker weitreichender Zusammenhänge.” (“Thus many souls are touched when misfortune befalls an outstanding discoverer of far-reaching connections.”) (Heubner, Eröffnungsansprache, Verhandlungen der DGP (14) 1938, p. 27 n.d.). Heubner also organized a card that was sent to Loewi in prison in Graz (Löffelholz and Trendelenburg 2008). Heubner makes it clear that he does not always agree with the policies propagated by the National Socialists with the words “[i]n Wahrheit sind ja diese beiden Eigenschaften des Menschen [gemeint sind Verstand und Redlichkeit] viel häufiger vereint, als es nach Propagandareden oder -zeitschriften zuweilen scheinen möchte” (“in truth, these two qualities of man [meaning intellect and honesty] are much more frequently combined than propaganda speeches or journals would sometimes make it seem”). At the end of his speech, he emphasizes “[u]nd gleichermaßen haben wir die Aufgabe, mit den Mitteln der logischen Beweisführung der Wahrheit zum Siege zu verhelfen, wo irgendwelche Nebeninteressen Schädigungen menschlicher Gesundheit durch giftige Einwirkungen zu behaupten oder abzuleugnen suchen” (“and likewise we have the task of using the means of logical reasoning to help the truth to triumph where any secondary interests seek to claim or deny damage to human health through toxic effects.”) Heubner’s conclusion to his speech is also striking. In complete contrast to Flury, he does not conclude with a hymn of praise for “the Führer” or the National Socialist goals but ends soberly with the sentence “Ich denke, wir alle werden in den kommenden Verhandlungen sowohl den Zug nach unserem Ankergrunde, wie auch das Spiel der Strömung von rechts und links empfinden.” (“I think that in the coming negotiations we will all feel both the pull towards our anchorage, and the play of the current from the right and the left.”) (Heubner, Eröffnungsansprache, Verhandlungen der DGP (14) 1938, pp. 28–29 n.d.).

### 15th session from August 22–24, 1947, in Hamburg

At the first conference after the war, which took place in Hamburg from August 22–24, 1947, two speakers with very different positions gave the opening speeches. These were the then First Chairman Behrend Behrens and the soon-to-follow First Chairman Otto Riesser, who was given the honorary chairmanship at this conference.

Behrens began his speech by welcoming those present, with a special greeting to honorary chairman Otto Riesser. He addressed a few words directly to Riesser himself: “Ich darf Ihnen versichern, daß, auch nach Ihrem Ausscheiden aus dem Vorstand, unsere Gesellschaft als solche niemals die Maßnahmen gebilligt hat, die der Nationalsozialismus gegen Sie ergriffen hat. Wir sind eine Vereinigung von Wissenschaftlern und als solche sind wir zu strenger Kritik an uns selbst und auch an anderen erzogen. Es ist deshalb in sich begründet, wenn die Wissenschaft und damit auch wir zur Partei schon von Anfang an in viele und grundsätzliche Widersprüche getreten sind.” (“I can assure you that, even after your departure from the board, our society as such has never approved of the measures taken against you by National Socialism. We are an association of scientists and as such we are trained to be severely critical of ourselves and others. It is therefore inherently justified if science, and thus we too, have entered into many and fundamental contradictions with the Party from the very beginning.”) (Behrens, Eröffnungsansprache, Verhandlungen der DGP (15) 1947, p. 1). In contradiction to this statement, Behrens goes on to honor the dead. He particularly emphasizes the late Ferdinand Flury, who was “the fatherly good spirit of the Society” (Behrens, Eröffnungsansprache, Verhandlungen der DGP (15) 1947, p. 2). As has already been discussed, Flury was an avowed National Socialist who had clearly expressed his political position at the previous conference in 1938. In contrast, the two members Emil Starkenstein (1884–1942), murdered in the Mauthausen concentration camp, and Hermann Freund (1882–1944), murdered in the Ausschwitz concentration camp, are only briefly mentioned, although both, like Riesser, were persecuted on racial grounds (Löffelholz and Trendelenburg 2008; Leopoldina errichtet Stele zum Gedenken an NS-Opfer 2009; Hermann Freund, (n.d.)).

Riesser, on the other hand, kept his opening speech very brief and to the point. He thanked those present for awarding him the honorary chairmanship and above all commemorated the deceased members who “were robbed of their lives” (Riesser, Eröffnungsansprache, Verhandlungen der DGP (15) 1947, p. 3). He emphasized: “Niemand, der nicht selbst das Los der Vertriebenen teilte, kann den Kummer und die seelische Not dieser aus ihrem Vaterland verjagten Menschen, niemand das Heimweh ermessen, das ihr schwerstes Leid war. Ihnen zu gedenken in kollegialer und herzlicher Anteilnahme, ist uns selbstverständliche Pflicht.” (“No one who has not himself shared the fate of the expellees can understand the grief and emotional distress of these people who were driven from their homeland, no one can fathom the homesickness that was their greatest suffering. It is our natural duty to remember them in collegial and heartfelt sympathy.”) (Riesser, Eröffnungsansprache, Verhandlungen der DGP (15) 1947, p. 3). Riesser does not mention Ferdinand Flury at all.

### The DGP’s dealings with Jewish members

Due to the lack of data from the National Socialist era, it is difficult to conduct a precise analysis of the DGP’s treatment of Jewish members. There are hardly any sources in which the political stance of the specialist society was explicitly stated. This means that there was also no publicly articulated resistance to the National Socialist persecution of Jewish members and other physicians.

When the National Socialists came to power in 1933, Jews made up 0.9% of the general population in Germany and 15–16% of the medical profession (Seidler 2000). The proportion of Jewish members of the DGP in 1933 is not known. From the membership list from 1931/32, 47 Jewish members can be identified. With a total membership of 260 members in that year, this corresponds to 18%, which is slightly higher than the proportion of Jews among doctors in general.

Erich Muscholl, who published his article “Founding history and the first 25 years of the German Pharmacological Society” in February 1995, reports on a board meeting on October 1, 1933, at which time the DGP board consisted of the members Ferdinand Flury, Otto Gros, Otto Riesser, Werner Lipschitz, Otto Loewi, and Edvard Poulsson. The minutes of the board meeting show the following: “In a very detailed discussion, Mr. Heubner pointed out that the Ministry was insisting on a change to the board and that if this was rejected, the society would be dissolved. Mr. Riesser noted that the majority of those present considered the resignation of the non-Aryan board members to be unavoidable. He proposes the following wording: ‘Under the present circumstances, the Pharmacological Society is not in a position to form its Board of Directors in accordance with the existing statutes. It therefore delegates the responsible leadership to Prof. Dr. Heubner until new statutes are drawn up.’ The version is adopted unanimously.” (Muscholl 1995).

As a result, the “non-Aryan” members Otto Riesser, Otto Loewi, and Werner Lipschitz were dismissed from the board (Löffelholz and Trendelenburg 2008). On October 11, 1933, Heubner informed the district court of the resignation of the board. However, the court insisted that there had to be two board members. Heubner reacted quickly with Janssen and thus prevented the dissolution of the DGP. The next general meeting took place in Göttingen on September 17, 1934 (Muscholl 1995). Around 30–40 participants were present, consisting only of “reichsdeutschen Nichtjuden” (“Reich German non-Jews”) (Heubner Tagebuch 10, 1932–1935), entry from September 17, 1934, ArchMHH, Dep. 13, 118).

At this general meeting, a number of amendments to the articles of association were also made, although these affected all associations at the time and not just the DGP in particular. The amendments to the statutes read as follows: “§ 7a The board requires the confirmation of the Reich Ministry of the Interior, § 7b The Reich Minister can dismiss board members at any time, § 7c The Reich Minister of the Interior has the right to suspend or revoke resolutions of the society’s board and § 14a Amendments to the statutes are subject to the approval of the Reich Minister of the Interior” (Muscholl 1995).

On October 22, 1935, Behrens proposed that “all members who have not paid dues since 1933 and whose mailings are returned as undeliverable should be removed from the list of members” (Verhandlungen der Deutschen Pharmakologischen Gesellschaft, 12. Tagung, gehalten in München vom 20.−23. Oktober 1935, ArchMHH, Dep. 13, 41). Behrens could only mean the members who had already emigrated. This exclusion requested by Behrens was rejected by the board (Muscholl 1995).

As Hellmut Weese points out in a letter to Prof. Bacq from Lüttich, members were never actually removed from the membership lists (Hellmut Weese an Zénon Marcel Bacq, Prof. am Pharmakologisches Institut der Universität Lüttich, Schreiben vom 23.2.^1948^, ArchMHH, Dep. 13,2).

If one compares the DGP membership lists still available in the society’s archives, Weese’s statement appears to be correct, at least for the period up to 1938. An analysis of the persecuted DGP members was carried out on the surviving membership lists for the years 1931/32, 1934/35, 1935/36, and 1936/38. The analysis was based on the work of Mispagel and Seifert (2025). However, 23 additional members persecuted by the Nazis were identified. These members will be analyzed in detail in a forthcoming study. Fifty-six persecuted DGP members are listed in total. With a few exceptions, these are listed consistently on all surviving membership lists. Only 17 of the 56 pharmacologists can no longer be found on the last surviving list before the outbreak of the World War II (1936/38), with six of the 17 pharmacologists having died. The reason for the absence of the other eleven remains unclear. However, it can be assumed that the absence could be due to difficulties in obtaining addresses due to emigration abroad. A deliberate exclusion of opponents of the regime and Jewish scientists on the part of the DGP is unlikely, as the majority of persecuted DGP members are still listed on the membership lists despite emigrating abroad, and the membership figures remain stable in the 1930s. For example, both Otto Loewi and Otto Krayer, who both emigrated abroad due to National Socialist policies in Germany, were still on the membership list in 1936/38. At this time, Krayer was already teaching abroad. Loewi had been dismissed from his post and his family was under threat in Germany (cf. Table 8). Weese’s statement cannot be verified in detail after 1938, as the next surviving list dates from 1949. However, it can be assumed that Weese’s statement is true, as the exclusion of non- “Aryan” members from the medical societies was generally completed by 1935 at the latest (Mitgliederliste der Deutschen Pharmakologischen Gesellschaft (1932); Mitgliederliste der Deutschen Pharmakologischen Gesellschaft (1936); Mitgliederverzeichnis der Deutschen Pharmakologischen Gesellschaft (1938); Mitgliederverzeichnis der Deutschen Pharmakologischen Gesellschaft (1936)).

**Table 8:**
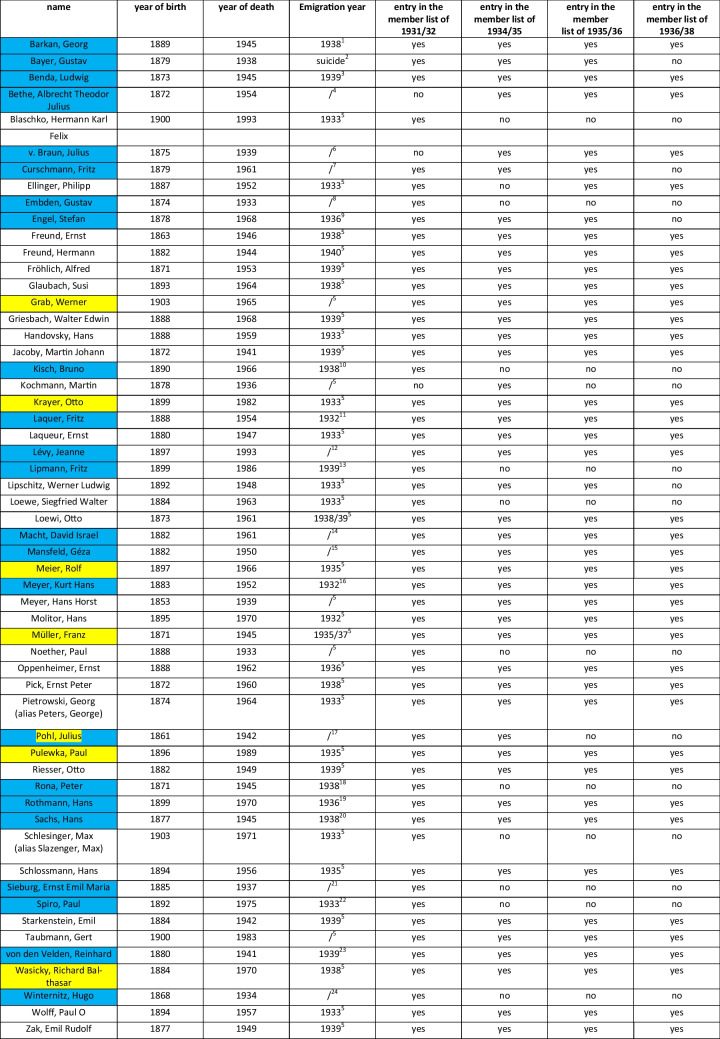
List of members of the DGP who were persecuted by the Nazis for racial or political reasons

Yellow marked: members who were persecuted by the Nazis for political reasons or because they were married to a “not-arisch” woman; blue marked: newly discovered Jewish members. **Data source** ^1^*Georg Barkan*. (n.d.). https://www.dgim-history.de/biografie/Barkan;Georg;1049. Accessed 18 Jul 2025. ^2^Bullock. (n.d.) *Denkstunde der Universitäten.* https://www.i-med.ac.at/mypoint/thema/716791.html. Accessed 18 Jul 2025. ^3^*Benda, Ludwig* (2025). https://www.lagis-hessen.de/pnd/117584967. Accessed 18 Jul 2025. ^4^Stahnisch, F. W. (2023). *Bethe, Albrecht*. https://www.deutsche-biographie.de/dbo011632.html#dbocontent. Accessed 18 Jul 2025. ^5^Mispagel, M., Seifert, R. (2024). Scientific, bibliometric and biographical analysis of 71 Jewish and dissident pharmacologists persecuted in Germany between 1933 and 1945. Naunyn-Schmiedeberg’s Arch Pharmacol. https://doi.org/10.1007/s00210-024-03645-z. ^6^*Julius von Braun (Chemiker)* (2024). https://de.wikipedia.org/wiki/Julius_von_Braun_(Chemiker). Accessed 18 Jul 2025. ^7^*Fritz*Curschmann. (2024).https://de.wikipedia.org/wiki/Fritz_Curschmann_(Mediziner*). Accessed 18 Jul 2025.* ^8^*Embden, Gustav.* (n.d.). https://frankfurt.de/frankfurt-entdecken-und-erleben/stadtportrait/stadtgeschichte/stolpersteine/stolpersteine-in-sachsenhausen/familien/embden-gustav. Accessed 18 Jul 2025. ^9^Stefan-Engel-Wissenschaftspreis (n.d.). https://www.dgspj.de/wp-content/uploads/forschung-stefanengelspreis-leben-2014.pdf. Accessed 18 Jul 2025. ^10^Walter, H. (1977). *Kisch, Bruno* in Neue Deutsche Biographie 11, S. 680-682 [Online Version]. https://www.deutsche-biographie.de/pnd11619328X.html#ndbcontent. Accessed 18 Jul 2025. ^11^*Laquer, Fritz* (2025). https://www.lagis-hessen.de/pnd/139618686. Accessed 18 Jul 2025. ^12^*Jeanne *Lévy. (2024). https://de.wikipedia.org/wiki/Jeanne_Lévy. Accessed 18 Jul 2025. ^13^*Curriculum Vitae Prof. Dr. Dr. Fritz Albert Lipmann* (n.d.). https://www.leopoldina.org/fileadmin/redaktion/Mitglieder/CV_Lipmann_Fritz_D.pdf. Accessed 18 Jul 2025. ^14^*David *Macht (2025). https://en.wikipedia.org/wiki/David_Macht. Accessed 18 Jul 2025. ^15^*Mansfeld *Géza (2024). https://hu.wikipedia.org/wiki/Mansfeld_Géza. Accessed 18 Jul 2025. ^16^Pummerer, R. (n.d.). *Kurt H. Meyer.* https://badw.de/fileadmin/nachrufe/Meyer%20Kurt%20H..pdf. Accessed 18 Jul 2025. ^17^Louven, A. (n.d.). Julius Pohl * 1861. https://www.stolpersteine-hamburg.de/?MAIN_ID=7&BIO_ID=1275. Accessed 19 Jul 2025. ^18^*Leopoldina errichtet Stele zum Gedenken an NS-Opfer.* (2009). Leopoldina. https://www.leopoldina.org/presse/pressemitteilungen/pressemitteilung/press/733/. Accessed 16 Dec 2024. ^19^*Hans Rothmann* (n.d.). https://www.dgim-history.de/biografie/Rothmann;Hans;1053. Accessed 18 Jul 2025. ^20^*Hans Sachs (Mediziner).* (2025). https://de.wikipedia.org/wiki/Hans_Sachs_(Mediziner). Accessed 18 Jul 2025. ^21^*Ernst *Sieburg. (2025). https://de.wikipedia.org/wiki/Ernst_Sieburg. Accessed 18 Jul 2025. ^22^*Paul Spiro*. (n.d.). https://spurensuche.dav-frankfurtmain.de/de/biografien/details/paul-spiro.html. Accessed 18 Jul 2025. ^23^*Reinhard von den *Velden. (2023). https://de.wikipedia.org/wiki/Reinhard_von_den_Velden. Accessed 18 Jul 2025. ^24^*Hugo *Winternitz. (2025). https://de.wikipedia.org/wiki/Hugo_Winternitz. Accessed 18 Jul 2025

The two members of the DGP, Otto Loewi and Otto Krayer, are briefly discussed below. Both were important pharmacologists of their time who emigrated abroad for different reasons.

### Otto Loewi

Otto Loewi was born in Frankfurt am Main on June 3, 1873. He studied medicine in Strasbourg and Munich from 1891 to 1896, where he became a great follower of Naunyn. Loewi decided to write his dissertation under Oswald Schmiedeberg before deepening his knowledge in the field of chemistry. From the winter semester of 1898, he was employed as an assistant and “practical physician” under Hans Horst Meyer (1853–1939) at the Pharmacological Institute at the University of Marburg. In 1909, Loewi was appointed full professor of pharmacology in Graz, although his Jewish confession initially caused difficulties. The highlight of his career was the award of the Nobel Prize for Physiology and Medicine on October 29, 1936, which Loewi received together with Sir Henry Dale (1875–1968) for the discovery of the chemical transmission of nerve effects. After the “Anschluss” (“Connection”) of Austria to the German Reich in March 1938, Loewi and his two sons were taken into “protective custody,” i.e., imprisoned in a concentration camp, for 2 months on the night of March 11–12 as part of a wave of persecution of prominent Jews. While he was still imprisoned, he was given a leave of absence and premature retirement. Before Loewi was able to emigrate to London at the end of August 1938, he had to transfer the money he had received for the Nobel Prize, which was still in a Swedish bank account, to the account of a National Socialist bank in the German Reich for Aryanization. Loewi fled to England completely penniless. In 1939, he moved to Oxford before receiving an invitation to New York, which he accepted. Despite his age of 67, Loewi built up a new, wide sphere of activity in the USA. After the war, he was lenient towards German pharmacologists, accepted honors, revived some of his friendships, but remained in the USA (Gier and Lembeck 1968; Lindner 1996; Otto Loewi, (n.d.); Fig. 3).

In 1955, the pharmacologist Ludwig Lendle wrote a letter to the board of the DGP calling for compensation for Loewi for the loss of the Nobel Prize money, as the state had not taken care of it. The board felt that a sum of money was inappropriate as compensation. Instead, it wanted to help Loewi claim his rights. They wanted to write a letter “to both the responsible German and Austrian authorities” [sic]. The board did not see any other way to help Loewi (Protokoll der Vorstandssitzung of September 5, 1955, ArchMHH, Dep. 13, 2). At the next board meeting, it was stated that Loewi had expressly requested that the DGP do nothing in the way of restitution (Protokoll der Vorstandssitzung of February 25, 1956, ArchMHH, Dep. 13, 2). Otto Loewi died in New York on December 25, 1961 (Lindner 1996).

### Otto Krayer

Otto Krayer was born on October 22, 1899, in Köndringen/Baden. After military service on the Western Front during the First World War, Krayer studied medicine in Freiburg, Munich, and Berlin from 1919 to 1924. He received his doctorate in 1926 and was employed by Paul Trendelenburg as an assistant in the pharmacology department in Freiburg. Together with Trendelenburg, Krayer moved to the Pharmacological Institute in Berlin in 1927, where he also worked as managing director from 1930 to 1932. In 1933, Krayer was appointed to the Chair of Pharmacology in Düsseldorf after it was to be filled due to Philipp Ellinger’s dismissal for racial reasons (Lindner 1996; Starke 2007). However, Krayer declined the appointment, which he explained in a letter dated June 15, 1933, as follows: “Sehr geehrter Herr Ministerialrat, […] Abgesehen von unwichtigen sachlichen Erwägungen war der Hauptgrund meines Zögerns der, daß ich die Ausschaltung der jüdischen Wissenschaftler als ein Unrecht empfinde, dessen Notwendigkeit ich nicht einsehen kann, da sie, wie mir scheint, mit außerhalb der Sphäre der Wissenschaft liegenden Gründen gestützt wird.” (“Dear Ministerial Councilor, […] Apart from unimportant factual considerations, the main reason for my hesitation was that I consider the elimination of Jewish scientists to be an injustice, the necessity of which I cannot understand, as it seems to me to be supported by reasons outside the sphere of science.”) (Starke 2007, p. 86).

With this decision, Otto Krayer was the only German pharmacologist to turn down a professorship that had become vacant due to the racial or political expulsion of the previous holder. In December 1933, Krayer emigrated first to Beirut and then to Boston in 1937. In Boston, he worked in pharmacology at Harvard University until his retirement in 1966 (Starke 2007). Otto Krayer died on March 18, 1982, in Tucson, Arizona (Lindner 1996; Fig. 3).

### How other specialist societies deal with Jewish members

To better understand how the DGP dealt with its Jewish members, other medical societies were analyzed for comparison. The German Society for Gynecology (DGG), originally known as Deutsche Gesellschaft für Gynäkologie, and the German Society for Pediatrics (DGfK), originally known as Deutsche Gesellschaft für Kinderheilkunde, were selected for this purpose. Both specialist societies had a comparatively high proportion of Jewish members at the time of National Socialism.

The German Society of Gynecology (DGG) was founded in Strasbourg in 1885. In 1931, the number of Jewish members of the DGG amounted to 170, which was about 20% of the total membership. By 1938, around 75% of the Jewish members had disappeared from the DGG’s membership lists. Only in a few cases is it known whether the withdrawal was voluntary or forced. After the National Socialists seized power in 1933, there was an increasing climate of “not being welcome” in the DGG, which presumably led to an increase in voluntary resignations by Jewish members. At the DGG conference in 1933, the then first chairman Walther Stoeckel (1871–1961) described the expulsion of Jewish members as a “necessary collateral damage in the interest of the recovery of the German people.” The first “Jew-free” conference was reported as early as October 1935 (Dross et al. 2016).

The first post-war congress took place in Karlsruhe on April 20, 1949. Jewish doctors now appear again in the list of great scientific deeds, but there is no mention of the fate they had to suffer. Whether contact was deliberately sought with the former persecuted members after the end of the Second World War cannot be traced. The fact is, however, that most of the more than 100 doctors who were ousted did not reappear in the DGG’s membership lists. The DGG did not issue a public apology to the persecuted Jewish members. Although some of them received honorary membership, leading Nation Socialists were also honored in this way in return (Dross et al. 2016).

At the 50th conference in 1994, the DGG issued a public apology to the victims of forced sterilization under National Socialism—there is no report of an apology to the expelled Jewish members (Dross et al. 2016).

The German Society of Pediatrics was founded in 1883. In 1933, around 30% of DGfK members were Jewish. Formally, Jewish members were not prevented from attending meetings even after 1933, but they were considered undesirable. Between 1933 and 1939, around 75% of the DGfK’s Jewish members resigned. In 1938, the secretary Fritz Goebel (1888–1950) finally removed the remaining Jewish members from the membership lists (Seidler 2000).

The first meeting after the war took place in 1948. At the second post-war meeting in 1949, Goebel endeavored to re-establish contact with Jewish members who had emigrated. Fritz Goebel was elected chairman of the society in the same year despite his clear anti-Semitic views [sic!]. There is also no sign of critical self-reflection among the pediatric supporters of National Socialism in the DGfK (Seidler 2000).

On October 3, 1998, a public memorial service was held as part of the DGfK’s annual conference for the colleagues expelled between 1933 and 1945, which can be seen as a public statement and a kind of apology (Seidler 2000).

### Public apology from the DGP for its treatment of Jewish members in the post-war period

A public apology from the DGP regarding its conduct towards Jewish members during the National Socialist era took almost 70 years in the making. During a symposium in honor of Emil Starkenstein’s (1884–1942) 130th birthday in November 2014, the then President of the DGPT, Prof. Dr. Ursula Gundert-Remy, made the following statement: “While preparing for this talk on the history of our Society, I became aware of facts I did not know before. I had to learn that in the past neither the German Pharmacological Society, of which Professor Starkenstein was a prominent member, nor the now DGPT had ever taken an official stance on events during the Nazi Regime. The Society had also never taken a position with regard to its own role in the unfortunate fate of the members of the Society who were removed from their posts at the university and elsewhere, had to leave their profession as scientists, had to leave their countries, lost their families and their own lives.”

I speak here on behalf of all members of the board and the Society when I declare that we deeply regret what happened during the Nazi Regime to members of our Society—particularly of Jewish origin.

I further declare that we are ashamed by the fact that in Nazi times, the Society and its board members failed to take morally appropriate action to defend and to protect its members—or at least to try to do so. We know that some of the members of the Society expressed their disapproval and some offered and provided help, in some cases with severe personal consequences—but this does not and cannot exonerate the board or the Society as a whole.

What happened cannot be undone. But we honor those who suffered by ensuring that they are not forgotten, and we will take every measure to ensure that such abhorrent events never occur again. (Gundert-Remy 2015).

## Conclusion

In summary, the German Pharmacological Society did not adequately address its past after World War II. Rather, its position was characterized by ambivalence and a lack of open discussion for decades about the damage the regime inflicted to the persecuted members.

The professional association showed inconsistent behavior in its dealings with Jewish members. It can be proven that between 1932 and 1938, there was no deliberate exclusion of Jewish members from the society. This distinguishes the DGP from other professional associations. For example, there is clear evidence of deliberate exclusion of Jewish members in the German Society for Gynecology. Nevertheless, in addition to explicit opponents of National Socialism, the ranks of the DGP also included very convinced National Socialists, foremost among them Ferdinand Flury, who clearly expressed his National Socialist views at the 14th conference in 1938.

However, Heubner’s speech at the same conference shows that this attitude was not the only one within the professional association. Heubner was never a member of the NSDAP, but according to Schagen (2008), he cannot be considered a “fundamental critic of the Nazi regime” either. Whether he was entrusted with the leadership of the society for a short time after the dissolution of the executive committee in 1933 precisely because he did not show any pronounced sympathy for Nazi racial ideology remains unclear.

Behrens also remains a difficult person to classify. Although it is known that he was a member of the NSDAP and was considered a supporter of Flury—as can be seen from the opening speech at the 15th conference in 1947—his political involvement remains unclear based on the available sources.

The deliberate appointment of Otto Riesser as the first chairman after the war suggests a moderate, personal attitude on the part of the re-established society—as well as a high degree of political calculation. This clear decision for Riesser was probably made easier because he saw himself as a Protestant and not as a Jew. Riesser’s return to Germany and his acceptance of the chairmanship should not be interpreted solely as a willingness and a good deed on his part.

Towards the end of the war, Riesser lived in complete poverty and presumably in hiding from the Nazis, surviving the war only by a series of coincidences (Hock 2024). He therefore had no choice but to accept the chairmanship to regain a foothold in Germany and save himself from his precarious situation. Another argument in favor of this is that although the Allies accepted the reestablishment of the society, they demanded that only untainted individuals—preferably returnees—should assume important positions.

In the early years, honorary memberships and Schmiedeberg plaques were increasingly awarded to Jewish members or to individuals who had been persecuted on racial grounds or had engaged in political resistance. The awarding of honorary membership to the expelled members Ernst Peter Pick, Otto Loewi, Otto Krayer, and Rolf Meier in particular shows the DGP’s attempt to honor emigrated pharmacologists for their services.

However, the desire to restore the international reputation of German pharmacology and strengthen its ties to the global scientific community of the discipline may also have played a role. As a result, some members who were supporters of National Socialism were also honored. For example, Ernst Frey (1878–1960), who held a chair in Göttingen and was a member of the Stahlhelm and the SA (BArch R 4901/13263 n.d.), was honored in 1952 together with Krayer. There can be no talk of a clear denazification or purge of society here.

Despite the destruction of many institutes, especially the Berlin Institute, pharmacologists quickly managed to reconnect with the international scientific community. A factor that should not be underestimated in this reconstruction of the DGP was the trust placed in it, especially by those members who had been persecuted and were conducting research abroad.

Nevertheless, for seven decades, the DGP joined in the collective silence about the behavior of its members during National Socialism. For example, Hans Herken (1912—2003), first chairman of the DGPT from 1962 to 1964, did not mention his own membership in the NSDAP in his book “Die Berliner Pharmakologie in der Nachkriegszeit” (Berlin Pharmacology in the Postwar Period). Even though an official apology from the DGP was announced in 2014, it comes 70 years too late. Other professional associations also dealt with National Socialism very late, but in this case, an apology was issued 20 years earlier. Why the DGP is lagging behind and is only now beginning to address its past remains unclear and clearly open to criticism.

## Data Availability

All source data for this study are available upon reasonable request from the authors.

## Abbreviations

DGG: German Society for Gynecology
DGP: German Society for Pharmacology
DGAI: German Society for Anesthesia and Intensive Care Medicine
DGfK: German Society for Pediatrics
DGPT: German Society for Experimental and Clinical Pharmacology and Toxicology e.V.
NSDAP: National Socialist German Workers’ Party
